# A New Description of Cellular Quiescence

**DOI:** 10.1371/journal.pbio.0040083

**Published:** 2006-03-07

**Authors:** Hilary A Coller, Liyun Sang, James M Roberts

**Affiliations:** **1** Department of Basic Sciences, Fred Hutchinson Cancer Research Center, Seattle, Washington, United States of America; **2** Molecular & Cellular Biology Program, University of Washington, Seattle, Washington, United States of America; **3** Howard Hughes Medical Institute, Fred Hutchinson Cancer Research Center, Seattle, Washington, United States of America; Harvard Medical SchoolUnited States of America

## Abstract

Cellular quiescence, defined as reversible growth/proliferation arrest, is thought to represent a homogenous state induced by diverse anti-mitogenic signals. We used transcriptional profiling to characterize human diploid fibroblasts that exited the cell cycle after exposure to three independent signals—mitogen withdrawal, contact inhibition, and loss of adhesion. We show here that each signal caused regulation of a unique set of genes known to be important for cessation of growth and division. Therefore, contrary to expectation, cells enter different quiescent states that are determined by the initiating signal. However, underlying this diversity we discovered a set of genes whose specific expression in non-dividing cells was signal-independent, and therefore representative of quiescence per se, rather than the signal that induced it. This fibroblast “quiescence program” contained genes that enforced the non-dividing state, and ensured the reversibility of the cell cycle arrest. We further demonstrate that one mechanism by which the reversibility of quiescence is insured is the suppression of terminal differentiation. Expression of the quiescence program was not simply a downstream consequence of exit from the cell cycle, because key parts, including those involved in suppressing differentiation, were not recapitulated during the cell cycle arrest caused by direct inhibition of cyclin-dependent kinases. These studies form a basis for understanding the normal biology of cellular quiescence.

## Abstract 2

Transcriptional profiling of fibroblasts induced to exit the cell cycle by distinct signals reveals distinctions and commonalities in the pathways to cellular quiescence.

## Introduction

Quiescence is the counterpart to proliferation: a reversible, nondividing state. Although it is a common state for many somatic cells, including stem cells, we know remarkably little about the regulation of cellular quiescence. For instance, is there just one kind of quiescent state, or do cells enter qualitatively different states depending upon the particular stimulus that initiated withdrawal from the cell cycle? Is cellular quiescence static, or is it a dynamic, continually changing physiological state? Is quiescence essentially a passive state (i.e., characterized by the absence of factors needed for cell division), or is it actively maintained?

Answering these questions has been difficult, because a quiescent cell is more notable for what it doesn't do (e.g., synthesize DNA, generate mitotic spindles) than for having readily identifiable traits of its own. One solution may be to use patterns of gene expression as a representation of the unique physiology of nondividing cells. Most of the earlier studies on fibroblasts have shown that there are substantial differences in the patterns of expressed genes in quiescent versus proliferating cells [1–6]. However, these previous studies did not distinguish gene-expression changes that were involved in quiescence, per se, from secondary responses induced by changes in the cellular environment. To circumvent this problem we used DNA microarrays to ask whether the same gene-expression changes occur when cells become quiescent in response to three independent growth-arrest signals, and for various periods of time. We found that fibroblast quiescence is a remarkably more dynamic and diverse collection of states than had been expected, but that it can also be described by a program of gene expression that is characteristic of multiple, independent quiescent states. This program of gene expression reveals the basic biological attributes of cellular quiescence.

## Results

### General Approach

**Figure 1:**
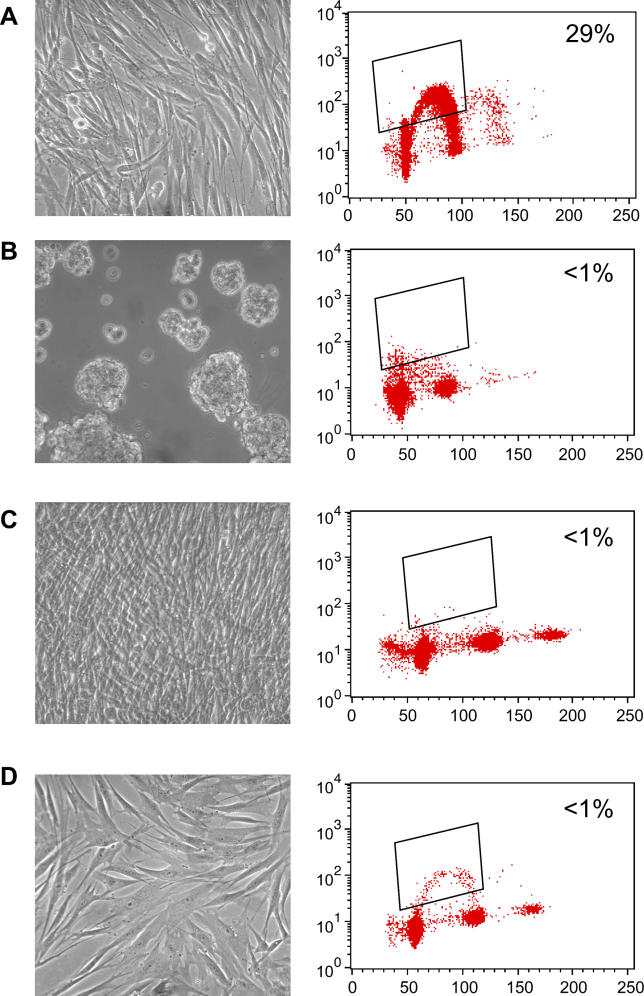
Model System: Growing and Arrested Cells Photographs of growing cells (A), and cells arrested overnight by loss of adhesion (B), contact inhibition (C), or mitogen withdrawal (D) are shown. Growing cells and cells that were arrested for 4 d by one of the three different arrest signals were incubated with bromodeoxyuridine (BrdU). Cell nuclei were labeled with an anti-BrdU antibody and propidium iodide, and analyzed by FACS. DNA content was plotted along the *x*-axis, and BrdU intensity was plotted along the *y*-axis. In growing cells, 28% of the nuclei incorporated BrdU during a 6-h incubation, while less than 1% of the arrested cells incorporated BrdU during the same time period. At 14 h <10% of the cells were in S phase. 2N (G1) cells in all conditions were purified by sorting.

Primary human fibroblasts were exposed to mitogen deprivation, growth at high cell density (“contact inhibition”), and disruption of cell-substratum adhesions (“loss of adhesion”) (Figure 1). Each of these three arrest protocols caused greater than a 95% decrease in the fraction of cells undergoing DNA synthesis (Figure 1). DNA microarrays were used to compare patterns of gene expression in growing cells and cells arrested for varying periods of time. By comparing three independent quiescence pathways, we were able to ask whether there were genes whose expression was associated with quiescence, per se, rather than with the particular mechanism of cell-cycle arrest. Our goal was to determine whether there is a novel set of genes that is associated with movement into and out of the cell cycle (G1 versus G0 [quiescent] cells) as opposed to the more commonly studied set of genes that is associated with progression through the cell cycle (see for instance [7]). We therefore excluded from our analysis the many genes whose expression is dependent upon the position of the cell within the cell cycle (e.g., S or M phase specific genes) by using a flow cytometer to collect only those with a 2N (G1) DNA content. Flow cytometry-based sorting was applied to all samples.

**Figure 2:**
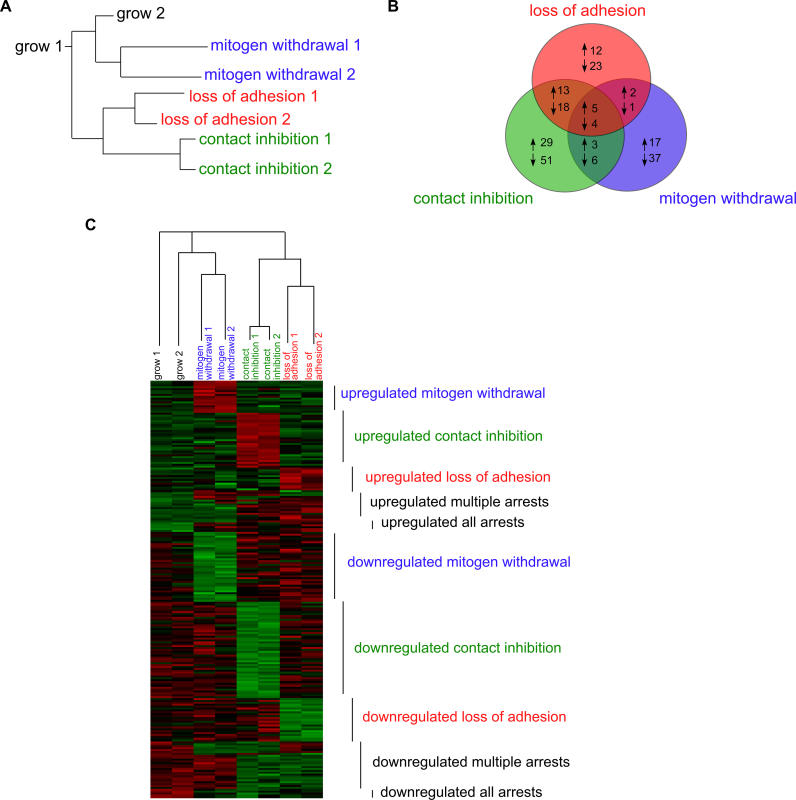
Quiescence Initiation (A) Neighbor-joining tree of quiescence initiation. Samples of growing cells and cells arrested by a single signal for 14 h were flow-sorted. Transcriptional distances (Affymetrix Genechip suite 4.0.) were determined for each pairwise comparison among samples as described in Materials and Methods. A matrix of transcriptional distances was used to generate a tree depicting the relationship among the growing and quiescent cells using neighbor-joining methods. (B) Venn diagrams depicting the number of genes regulated by one, two, or three arrest signals during quiescence initiation. The number of genes that were regulated by a single signal, by two out of three signals, or by all three signals was determined based on either consistent 2-fold-->regulation or “template-matching.” The number of genes expected to be downregulated by a single signal by chance is 7.8, and the number observed was statistically significant for all three arrest methods (*p* < 0.001). The number of genes expected to be upregulated by a single signal by chance is 5.1. The signature for genes upregulated by mitogen withdrawal or contact inhibition alone is significant at *p* < 0.001, while the signature for genes upregulated by loss of adhesion is significant at *p* = 0.004. One gene was expected to be downregulated by two out of three signals by chance. For mitogen withdrawal combined with contact inhibition, and loss of adhesion combined with contact inhibition, the observed number of downregulated genes was statistically significantly higher than expectation (*p* < 0.001). For mitogen withdrawal combined with loss of adhesion, the result was not statistically significant. The expected number of genes upregulated by two arrest signals is 0.75. For genes upregulated by mitogen withdrawal combined with contact inhibition, and by loss of adhesion combined with contact inhibition, the number of regulated genes is significant (*p* < 0.001). For mitogen withdrawal combined with loss of adhesion, the result was not statistically significant. The number of genes expected to be downregulated by all three signals was 0.85, and the observation of four was significant at *p* = 0.009. The number of genes expected to be upregulated by all three arrest signals is 1.2, and the observation of five is significant at *p* = 0.006. (C) “Heat maps” of genes regulated during quiescence initiation. Samples of growing cells and cells arrested by a single signal for 14 h were flow sorted and analyzed with microarrays. Genes regulated by one, two, or all three signals were identified both by consistent 2-fold change and by “template-matching” as described in Materials and Methods. Genes are represented by rows; columns indicate samples. The average difference values for each gene were normalized. The relative expression levels for a gene among the samples are indicated by green for a low value and by red for a high value. The figure was generated with Java Treeview [54]. The dendrogram above the figure depicts the topology of a neighbor-joining tree generated from the transcriptional distances between each sample. The complete tree is shown in (A).

To get an overview of the relationships among the different quiescent states, we measured the “genome-wide transcriptional distance” between a pair of samples, which was calculated by summing the differences in expression levels between the samples for all genes on the microarray [8]. A tree representing the relationships among cells arrested by different methods based on this measure is shown in Figure 2A. Note that independent, duplicate samples of cells arrested by each of the three methods are next to each other, demonstrating that cells arrested by different methods execute reproducible and signal-specific gene-expression changes.

We also used two methods to identify specific genes regulated in quiescent cells. In the first, we ranked the genes based on their similarity to a stereotyped example of expression changes, and used permutation tests to determine the extent of correlation expected by chance. In the second, we identified genes regulated 2-fold when cells became quiescent. The expected number of false positives was determined by permutation testing. In addition to determining the number of such genes, we visualized these genes in the relevant samples with “heat maps” [9] in which low expression of a gene in a given sample is indicated by green while high expression is indicated by red.

### Quiescence Initiation

Cells were exposed to arrest signals for 14 h, at which time the fraction undergoing DNA synthesis had decreased 70%–85%. The relatedness tree (Figure 2A) showed that the transcriptional profile of each quiescent cell population was reproducibly distinct from proliferating G1 cells. Thus, quiescence is not a prolonged pause in G1 but rather is characterized by distinct gene expression changes. Indeed, a significant number of genes (between 10 and 60) were uniquely upregulated or downregulated in cells exposed to each arrest signal for 14 h (Figure 2B and 2C). Some of the genes regulated by only one or two arrest signals relate to two central features of quiescent cells—decreased proliferation and decreased biosynthetic activity. The former include genes involved in mitogenic signal transduction and in pathways that directly regulate the cell cycle, and the latter include genes involved in lipid, nucleotide, and carbohydrate metabolism (Table 1). We concluded that each signal induced a unique quiescent state, because these fundamental features of quiescence (cessation of growth and division) are realized, at least in part, by different signal-dependent changes in gene expression. These included upregulated as well as downregulated genes, showing that quiescence is an active state.

**Table 1:**
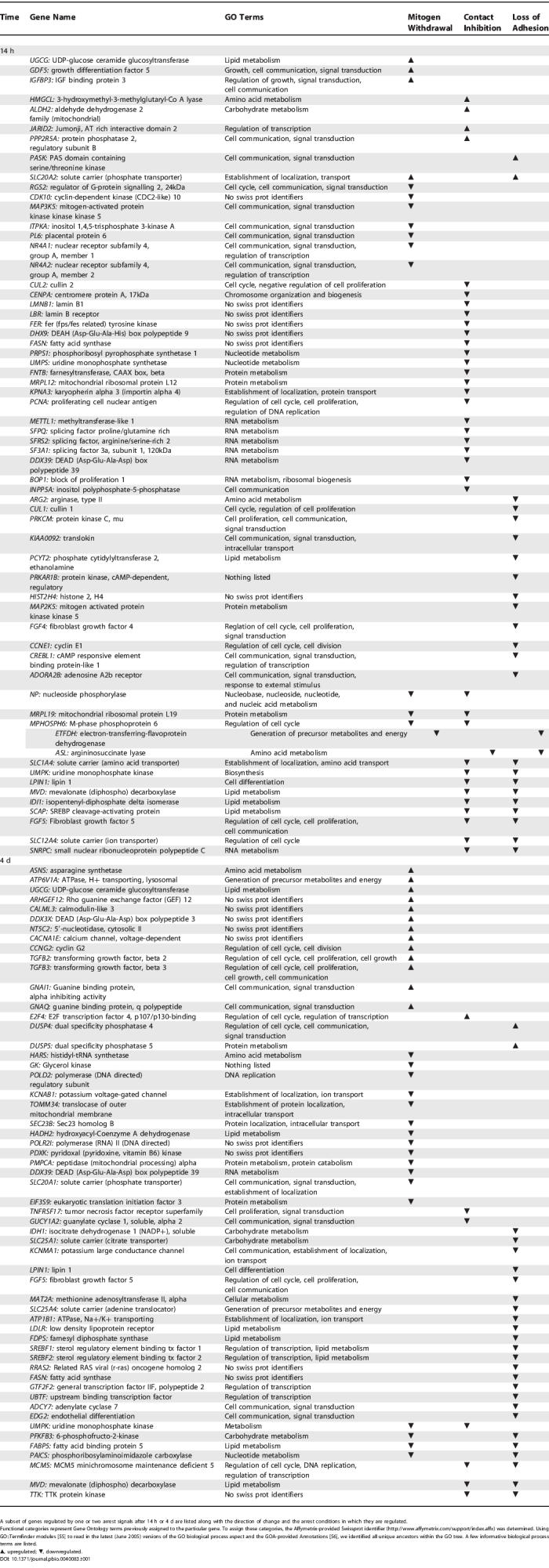
Genes Regulated by Specific Arrest Signals

There was a smaller but statistically significant set of genes that was regulated by all three arrest signals. The upregulated genes included three transcription factors, *ZNF124, HES1,* and *RB1,* and downregulated genes included the transcription factor *GABPB2,* the mitotic regulator *MAD2L1,* and *VEGFC*. The fact that four pleiotropic transcription factors were regulated during entry into quiescence suggested that the cells might induce a generalized program of quiescence gene expression that would be manifest at later times.

### Quiescence Maintenance

**Figure 3:**
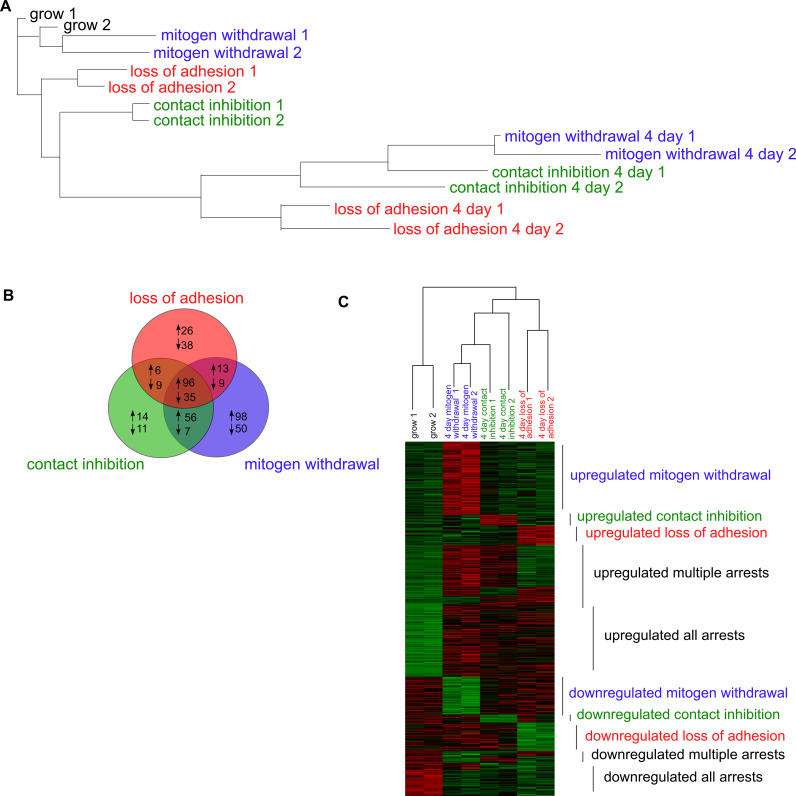
Quiescence Maintenance (A) Neighbor-joining tree of quiescence initiation and maintenance. Neighbor-joining tree of the transcriptional distance between samples after overnight and 4 d of quiescence generated as described in Figure 2A. (B) Venn diagrams depicting the number of genes regulated by one, two, or three arrest signals during quiescence maintenance. Methods are as described in (Figure 2B), except that cells were arrested for 4 d. The number of genes downregulated specifically by mitogen withdrawal or by loss of adhesion was significantly greater than the expectation (7.8 genes) at *p* < 0.001. The number of genes downregulated by contact inhibition was not statistically significant. The expected number of genes upregulated by a single signal was 5.1, and the signature for genes upregulated by each of the signals individually was statistically significant (*p* < 0.001). The expected number of genes downregulated by two signals was one, and the observed values for all three combinations were significant at *p* < 0.001. The expected number of genes downregulated by all three arrest signals was 0.85, and the observed value of 33 was highly significant (*p* < 0.001). All combinations of upregulation by two signals were statistically significantly elevated (*p* < 0.001) as compared with the expectation of 0.75 genes. The number of genes expected to be upregulated by all three signals was 1.2, and the observed value of 96 was significantly different (*p* < 0.001). (C) “Heat maps” of genes regulated during quiescence maintenance. “Heat map” of the genes regulated after 4 d of quiescence and dendrogram are as described in Figure 2C.

We next asked whether the immediate response to arrest signals, which was characterized largely by signal-specific changes, foreshadowed mostly signal-specific expression changes at a later time point. Alternatively, did these short-term largely divergent states ultimately coalesce into a common quiescent state? After 4 d, there were many more expression changes than after an overnight arrest, and the transcriptional distance to each state increased on average 3.3-fold (Figure 3A). Moreover, each arrest state maintained its unique character. There continued to be strong signatures for genes that were regulated specifically by a single arrest signal (Figure 3B and 3C), and many of these genes were regulators of cell growth and cell division (Table 1).

We wanted to ascertain whether these quiescence state-specific expression changes reflect bonafide differences in the gene expression pattern in response to different signals, or whether they might reflect only a difference in the kinetics of the induction of a similar response induced by different signals. We discovered that much of the signal-specific signature observed after 14 h persisted through 4 d (Figure S1). This makes it unlikely that differences reflect simply timeshifting of a common signature. We also discovered a surprisingly strong signature for genes upregulated by mitogen withdrawal and downregulated by loss of adhesion, or vice versa, at both quiescence initiation and quiescence maintenance (Figure S2). For these reasons, we concluded that the gene expression changes observed most likely reflect qualitatively different quiescent states and do not represent the same gene expression changes induced by different signals with different kinetics.

**Figure 4:**
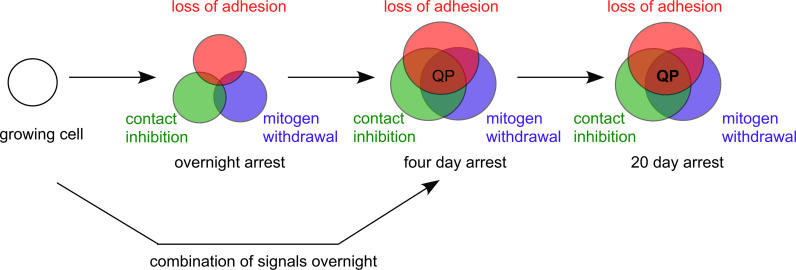
Summary of the Relationships among Quiescent States In cells arrested overnight by a specific arrest signal, gene expression changes are largely signal-specific with a small amount of overlap. When cells have been arrested for 4 d, there continue to be signal-specific changes, but now there are a large number of commonly regulated genes that we refer to as a “quiescence program.” In cells arrested for 20 d, the intensity of the changes in the quiescence program are magnified. The three circles are intended to reflect the enhancement of the quiescence program at 20 d. We have not analyzed all the three arrest conditions at this late time point. When cells are arrested by a combination of extracellular signals, quiescence program gene expression changes appear after only an overnight arrest.

Superimposed on this signal-specific pattern, however, was also a convergence of gene expression changes. After 4 d of arrest, 35 genes were downregulated and 96 were upregulated by all three signals (Figure 3B and 3C). These results suggested that there might be a fibroblast “quiescence program,” a set of gene-expression changes central to any long-term quiescent state and independent of the specific arrest signal (Figure 4).

### Definition of a Quiescence Program, and Its Expression during Prolonged Arrest

There were many gene-expression changes that were shared by cells reaching a longer transcriptional distance from growing cells—those arrested by an individual signal for 4 d and those grown to confluence and maintained for 4 d or 20 d (see below). We defined a “quiescence program” (see Materials and Methods) by identifying genes consistently regulated in these deeper arrests. There were 116 upregulated and 33 downregulated genes that met these criteria, while the expectation due to random chance was approximately 1 (Table 2). The quiescence program included not only genes involved in regulating cell growth and division, but also genes that suppressed apoptosis, suppressed differentiation, and were involved in intercellular communication; all of these are likely to be essential to maintaining cells and tissues in a reversible, viable arrested state (see Discussion).

**Table 2:**
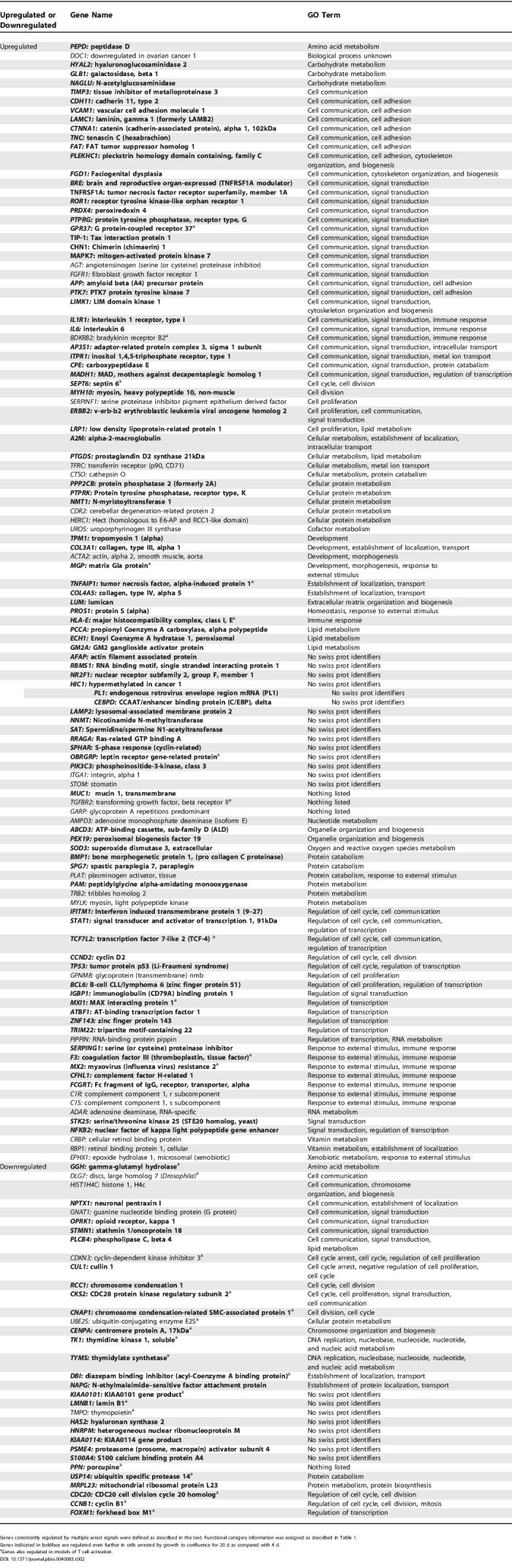
Genes in the Quiescence Program

**Figure 5:**
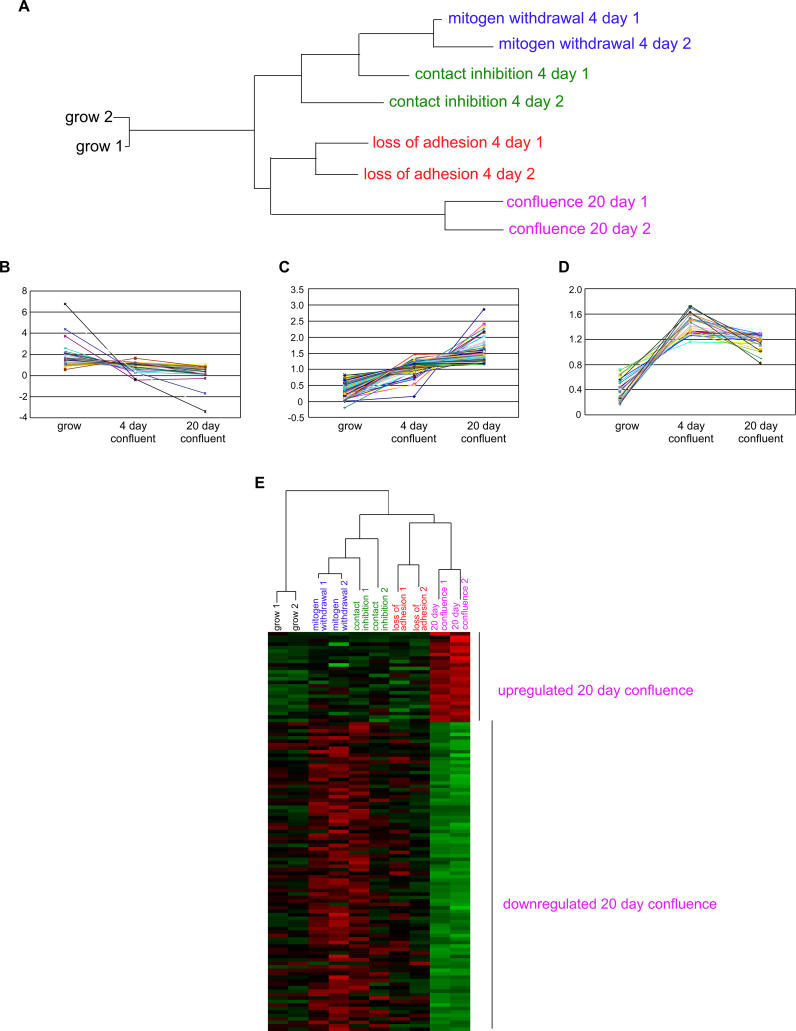
Change in Expression with Longer Arrest Time (A) Neighbor-joining tree defining the relationship between growing cells, cells arrested by mitogen withdrawal, contact inhibition, or loss of adhesion for 4 d, and by growth to confluence for 20 d. Transcriptional distances (Genechip suite 4.0) were determined and used to construct the distance matrix and neighbor-joining tree as described in Figure 2A. (B–D) Plot of expression levels in quiescence program genes in growing, 4-d confluent and 20-d confluent cells. Mean expression values in growing cells, cells arrested by growth to confluence for 4 d and cells arrested by growth to confluence for 20 d were normalized by dividing by the average value. The data are plotted for (B), all downregulated quiescence program genes; (C), the 89 upregulated quiescence program genes that were more strongly upregulated at 20 d than 4 d; and (D), the 26 upregulated quiescence program genes that were more strongly upregulated at 4 d than 20 d. (E) Heat maps of gene expression changes characteristic of longer arrest. Gene expression patterns for genes downregulated or upregulated in 20-d confluent cells but not cells arrested by mitogen withdrawal, contact inhibition, or loss of adhesion for 4 d are shown. Heat maps were generated as described for Figure 2C. The expected number of genes, 1.3 upregulated genes and 0.5 downregulated genes, were determined by permutation testing. The observed values of 26 and 87, respectively, are highly significant (*p* ≪ 0.001).

Previous studies had shown that cells deprived of serum for weeks rather than days took longer to reenter the cell cycle [10,11]. At the molecular level this might be manifest as an even greater change in the expression of those genes that had been regulated during shorter arrests. We allowed the cells to grow to confluence and then maintained them in an arrested state with regular medium changes for approximately 20 d. We compared them with cells arrested by the same protocol for only 4 d. Prolonged quiescence resulted in the longest transcriptional distance from growing cells of any arrested state (Figure 5A). There was a 50% increase in the transcriptional distance to growing cells in cells arrested by growth to confluence for 20 d versus 4 d. Notably, 82% (27/33) of the downregulated genes and 77% (89/116) of the upregulated genes in the quiescence program were repressed or induced more strongly at 20 d of quiescence than at 4 d (Figure 5B–5D). Thus, arrest for longer periods of time, which results in a “deepening” of the quiescent state, is associated with an exaggeration of the quiescence program (Figure 4).

Prolonged arrest by growth to confluence also resulted in a signal-specific gene expression profile in addition to an enhancement of the quiescence program. We identified 26 upregulated and 87 downregulated genes that were consistently regulated in cells grown to confluence for 20 d but not by mitogen withdrawal, contact inhibition, or loss of adhesion for 4 d (Figure 5E and Table 3).

**Table 3:**
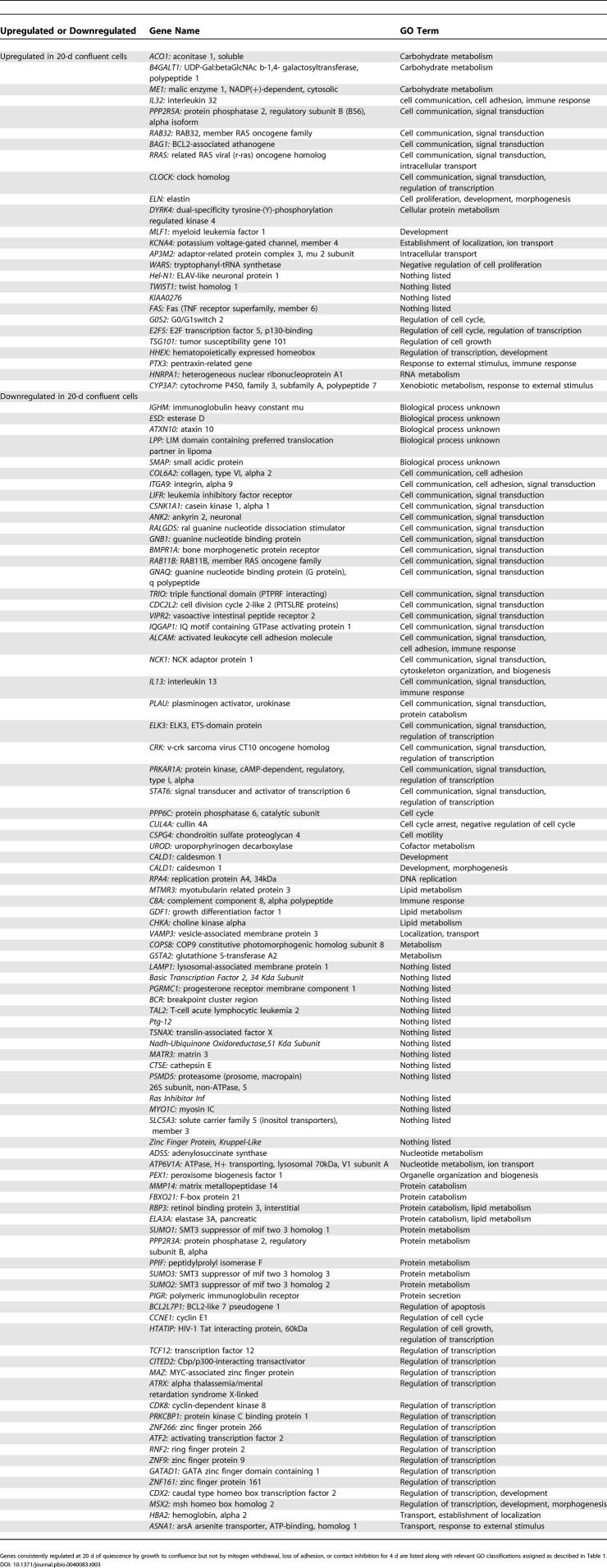
Genes Regulated by Quiescence for Longer Periods of Time

### Combination of Arrest Signals; Rapid Onset of Quiescence

**Figure 6:**
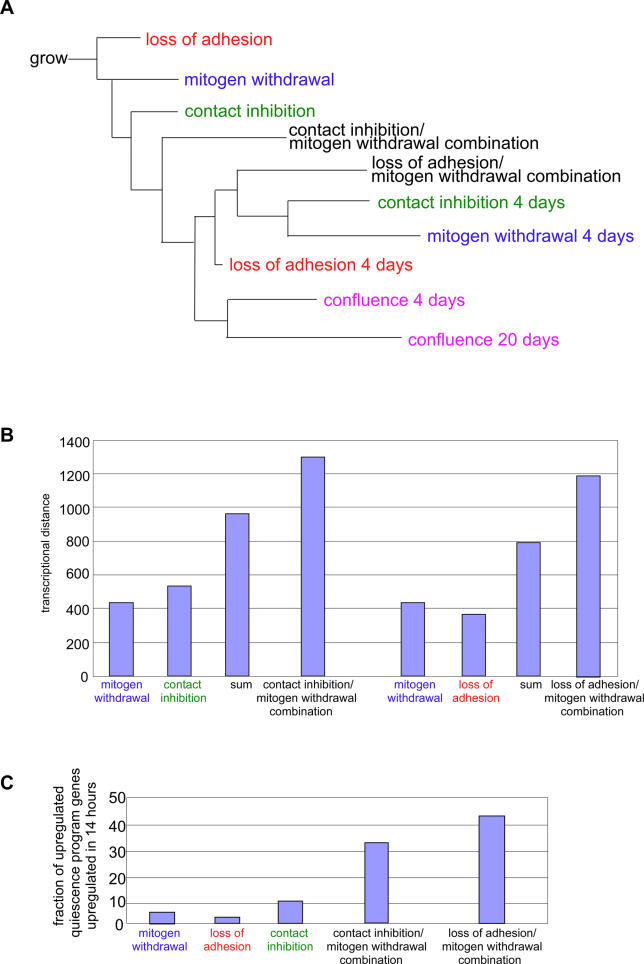
Combination of Arrest Signals Results in Synergistic Effects (A) Neighbor-joining tree defining the relationship among growing cells, cells arrested by a single signal overnight, by a combination of signals, by single signals for 4 d and by longer arrest. Transcriptional distances (Genechip suite 4.0) were determined and the mean transcriptional distances for the two examples of each arrest condition were used to construct the distance matrix and neighbor-joining tree. (B) Mean transcriptional distance from growing cells to cells arrested by individual signals overnight, and the sum of the distances, is plotted, and compared with the transcriptional distance from growing cells to cells arrested overnight by a combination of signals. (C) The fraction of upregulated quiescence program genes that were upregulated by each individual signal and by combinations of signals after an overnight arrest.

Fibroblasts are most effectively made quiescent by exposing them to combinations of arrest signals. We hypothesized that a molecular explanation might involve activation of the quiescence program. To test this, we arrested cells for 14 h by withdrawing mitogens and simultaneously either plating them at high density or arresting them by loss of adhesion. Manipulating two pathways together had a synergistic effect; cells arrested by either combination were even farther from growing cells than the sum of the distances to the individual arrest states (Figure 6A and 6B).

The fraction of upregulated quiescence program genes that were also 2-fold upregulated in cells arrested overnight by a single signal was small—5% for mitogen withdrawal, 9% for contact inhibition, and 3% for loss of adhesion. In contrast, a much higher fraction of the upregulated quiescence program genes were regulated in cells arrested overnight by a combination of signals—32% for mitogen withdrawal plus contact inhibition, and 43% for mitogen withdrawal plus loss of adhesion (Figure 6C). Therefore, combining arrest signals shortcircuited the pathway to cellular quiescence, resulting in a more rapid expression of the quiescence program (Figure 4).

### Cell-Cycle Arrest by Overexpression of Cyclin-Dependent Kinase Inhibitors

The observed induction of quiescence program genes might have been a consequence of cell-cycle arrest. For instance, different arrest signals might all directly downregulate cell-cycle proteins, and expression of quiescence program genes would then be a downstream, secondary effect. Alternatively, expression of the quiescence program could be the primary event that is induced in common by each of the extracellular anti-proliferative signals. Cell-cycle arrest would be one downstream effect of the quiescence program, being induced by a subset of the quiescence program genes.

**Figure 7:**
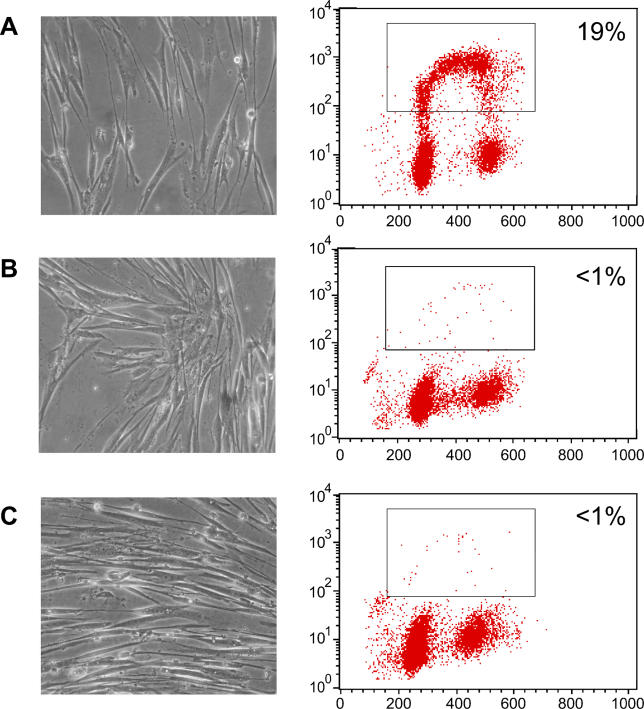
Cells Arrested by CKI Overexpression Photographs of cells transduced with an empty vector (A), or a vector encoding p21 (B), or p27 (C). Methods and axes are as in Figure 1. Tetraploid cells were gated out. In cells transduced with an empty vector, 19% of the nuclei incorporated BrdU, while less than 1% of cells expressing p21 or p27 incorporated BrdU during the same time period.

To distinguish between these models, we bypassed extracellular signal transduction pathways, and directly arrested the cell cycle by inhibition of cyclin-dependent kinases (CDKs). The first model predicts that cell-cycle arrest should be sufficient to induce the quiescence program, while the second predicts that it would not. We used retroviral-mediated gene transduction to ectopically overexpress the CDK inhibitors (CKI) p21 or p27. This efficiently blocked cell proliferation, and did not induce cellular senescence as monitored by expression of senescence-associated β-galactosidase (Figure 7).

**Figure 8:**
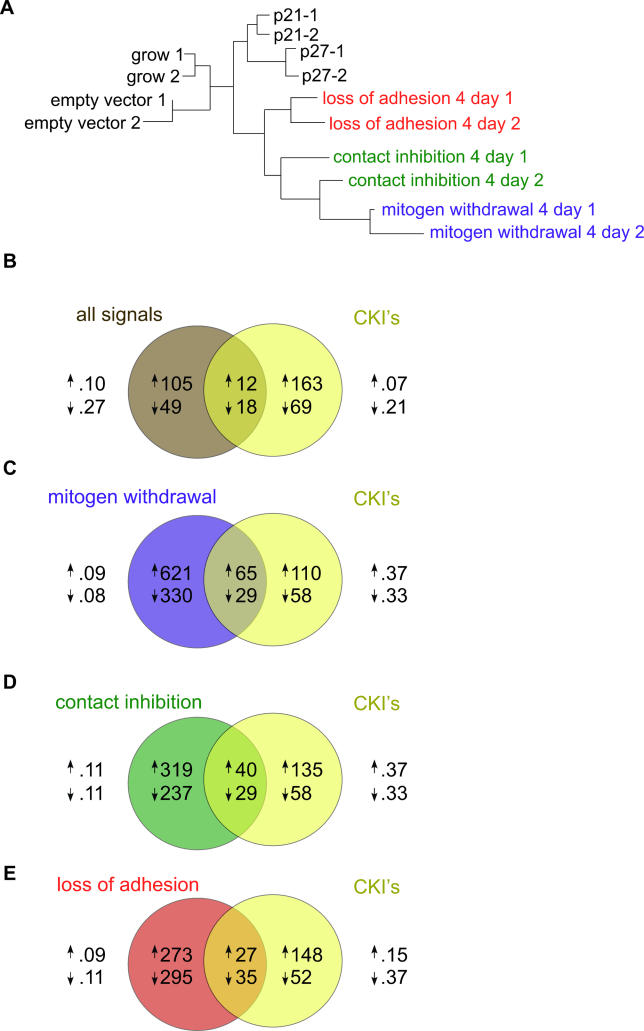
CKI Overexpression Does Not Closely Resemble the Quiescent State Induced by Any Extracellular Signal (A) Neighbor-joining tree of growing cells, cells arrested by extracellular signals for 4 d, cells transduced with an empty vector, and cells transduced with a CKI. Transcriptional distances were determined for all pairwise comparisons (Genechip suite 5.0), and neighbor-joining trees were drawn as described in Materials and Methods. (B–E) The overlap between genes regulated by CKI overexpression and genes consistently regulated by all signals (B), mitogen withdrawal (C), contact inhibition (D), or loss of adhesion (E) was determined. The number of regulated genes is shown within the circle. The fraction of regulated genes within the overlap area is provided outside the circles. Genes consistently downregulated by all three arrest signals were most likely to be regulated by CKI overexpression.

Cells arrested by CKI overexpression formed a clade that was distinct from the clade comprised of cells arrested by extracellular signals (Figure 8A). The transcriptional pattern induced by CKI overexpression did not closely resemble the quiescent state induced by any particular extracellular signal. Only a small proportion of all of the genes regulated by all signals or an individual signal were also regulated by CKIs, with the greatest overlap among genes downregulated by all signals (Figure 8B–8E). Indeed, although downregulated quiescence program genes were likely also to be downregulated by CDK inhibition, many upregulated genes in the quiescence program were not induced by CDK inhibition. More precisely, genes regulated by four different anti-proliferative signals were extremely likely (78%) to be regulated in a fifth condition. Among genes that were consistently downregulated 2-fold in all of the quiescent states, a significant fraction (54%) was also downregulated in cells arrested by p21 and p27 (Table 3). These commonly regulated genes clustered into the functional category of cell-cycle regulators. Thus, there was a self-reinforcing component to cell-cycle arrest, in the sense that CDK inhibition caused downregulation of additional genes that also directly affected the cell cycle.

In contrast, only 12% of the quiescence program genes that were upregulated 2-fold in all arrest conditions were also upregulated by CKI overexpression, and these genes did not fall into any particular functional class (Table 2). Therefore, a single molecule that causes cell-cycle arrest could not recapitulate the complete, functionally diverse quiescence program.

### Quiescent Cells, but Not Cell Cycle Arrested Cells, Are Resistant to Differentiation

**Figure 9:**
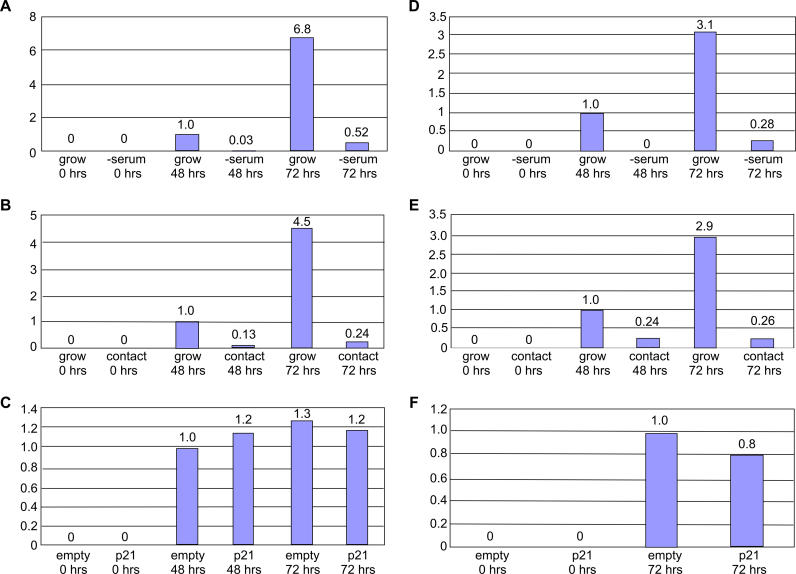
Quiescence, but Not Simply Cell-Cycle Arrest, Protects against Differentiation Human dermal fibroblasts 91-SF5 were transduced with a MyoD-estrogen receptor fusion, selected for stably transduced cells, and differentiated. At the indicated time points after differentiation, myogenin (A–C) and myosin heavy chain (D–F) expression levels were determined with real time RT-PCR using GAPDH as an internal standard and normalized values were plotted. Myogenesis marker expression induced by differentiation was inhibited in cells that were made quiescent for 4 d by serum withdrawal (A and D) or contact inhibition (B and E) as compared with growing cells. In contrast, in cells arrested by overexpression of the CKI p21, there was no reduction in myogenesis marker expression as compared with control cells transduced with an empty vector (C and F).

Reversibility is the defining characteristic that distinguishes quiescence from other nondividing cell states. Based on our gene expression results, we hypothesized that quiescent cells remain in this reversible state by activating pathways that prevent the inappropriate adoption of nondividing states associated with irreversible cell-cycle arrest, such as senescence and terminal differentiation. To test this hypothesis, we asked whether quiescent fibroblasts were resistant to terminal differentiation. We transduced human dermal fibroblasts (strain 91-SF5) with a retroviral vector expressing a MyoD-estrogen receptor fusion protein [12] and subsequently treated the cells with β-estradiol (10^−7^ M) to activate MyoD and induce the program of gene expression that results in myogenic differentiation [13], (see Materials and Methods for details). Muscle differentiation was monitored by real-time RT-PCR for expression of the myogenesis markers myogenin and myosin heavy chain. As can be seen in Figure 9, activation of MyoD in proliferating cells dramatically induced myogenin (Figure 9A) and myosin heavy-chain expression (Figure 9D), whereas fibroblasts that were made quiescent for 4 d by either serum withdrawal (Figure 9A and 9D) or contact inhibition (Figure 9B and 9E) were resistant to differentiation, as evidenced by an approximately 10-fold decrease in both myogenin and myosin heavy-chain expression levels. Quiescence did not affect MyoD-ER expression (unpublished data).

Our results had indicated that direct inhibition of CDKs arrested the cell cycle, but did not induce the quiescence program of gene expression. We therefore hypothesized that CKI-arrested cells would not share the same functional properties as naturally quiescent cells. Specifically, CDK inhibition did not induce the expression of genes that were associated with inhibition of differentiation (see Discussion), and we therefore tested their ability to undergo MyoD-induced myogenic differentiation. Fibroblasts expressing the MyoD-ER fusion were transduced with a retroviral vector expressing the CKI p21^Cip1^, or with an empty vector as a control. The p21 expressing cells stopped proliferating, whereas the control cells did not (unpublished data). After 3 d, cells were induced to differentiate by exposure to β-estradiol. As shown in Figure 9C and 9F, nonproliferating cells transduced with the CKI p21 and control cells expressed similar amounts of myogenin and myosin heavy chain. Thus, the cell-cycle arrest induced by p21 was compatible with myogenesis, which was in contrast to the differentiation resistance exhibited by fibroblasts that were quiescent due to contact inhibition or growth-factor deprivation. This supports our conclusion that the quiescence program of gene expression, which is induced in cells that become quiescent in response to external signals but not by direct CDK inhibition, ensures the reversibility of the quiescent state by inhibiting terminal differentiation.

## Discussion

The conceptually important points that emerge from our work are: 1) that quiescence is not a unique state but rather a collection of states that are determined by the initiating signal; 2) that it is possible to identify genes that are universally characteristic of quiescence (i.e., signal independent) implying the existence of a genetic program of quiescence; and 3) that quiescence is functionally different from cell-cycle arrest.

### Quiescence Is Not a Unique State

Our first major observation was that different quiescence signals arrested growth and division by different mechanisms, and on this basis we concluded that cells enter different signal-dependent quiescent “states.” The unique features of these different quiescent states were evident both in the pathways by which cells exited the cell cycle in response to antimitogenic signals, as well as in the pathways by which growth and proliferation were suppressed after the cells had entered the nondividing states. More specifically, we observed that different antiproliferative signals initiated cell-cycle exit by affecting the expression of different growth regulatory genes (Table 1). For example, contact inhibition resulted in downregulation of *cul-2, PCNA, lamin B* and its receptor, *CENPA,* biosynthetic enzymes in nucleotide metabolism, splicing factors, and different members of the inositol triphosphate signaling pathway. Loss of adhesion caused downregulation of *cyclin E1, Cul-1,* a protein kinase C isoform, another MAP kinase, and *FGF4*. Mitogen withdrawal caused the downregulation of G-protein–signaling molecules, and kinases from the MAP kinase and inositol-triphosphate–signaling pathways, as well as upregulation of *IGFBP3*. The importance of all of these proteins for controlling cell proliferation has been established, but their unique relationships to specific quiescence signals were not known. We do not mean to imply that the altered expression of any one gene alone would be sufficient to explain the mechanism of cell cycle exit in response to any particular anti-mitogenic signal. We suggest that it is the combinatorial impact of these multiple gene expression changes that defines the unique features of each quiescent state.

Interestingly, many of the genes that initially responded in a signal-specific manner during the initiation of quiescence became more uniformly regulated, in a signal-independent manner, after cells had been quiescent for 4 d. Many of these genes ultimately made up part of the quiescence program and are discussed below. Nevertheless, even at this late time point each anti-proliferative signal continued to maintain cells in unique quiescent states as evidenced by the regulation of new signal-specific genes that suppressed growth and division in different ways (Table 1). Thus genes essential for DNA synthesis *(POLD2),* RNA synthesis *(POLR2I),* and protein synthesis *(EIF3S9, HARS)* were uniquely downregulated by mitogen withdrawal after 4 d of quiescence. Many genes essential for various aspects of intermediary metabolism were uniquely downregulated after 4 d of loss of adhesion, including genes crucial for lipid, carbohydrate, and nucleotide metabolism (Table 1). Moreover, anti-proliferative pathways uniquely upregulated in mitogen-starved quiescent cells included those governed by *TGF-beta 2* and *-3*. In contrast, loss of adhesion induced the phosphatases *Dusp4* and *-5* (Map-kinase phosphatases), which are inhibitors of the MAP-kinase signaling pathway, whereas contact inhibition induced the quiescent cell-specific transcription inhibitor *E2F4*.

One implication of our work is that it will be important to consider the specific quiescent state from which cells are emerging when analyzing the effects of particular proteins on cell proliferation. It will be of interest to determine which particular quiescent states are found in vivo, as this should provide new insights into the mechanisms that control cell dynamics and tissue homeostasis during embryonic development and in mature animals. We speculate that the tissue-specific effects of particular oncogenes and tumor suppressors might be understood in terms of their functional relationship to different quiescent states.

### Quiescence Program

Superimposed on this diversity of states is a set of transcriptional responses that is shared by all quiescent fibroblasts. While on average only 9% of genes are regulated in any given arrest state, 78% of those regulated in four transcriptionally distant arrest states were also regulated in the fifth. Since these changes in gene expression are characteristic of quiescence per se, rather than of a particular mechanism of cell cycle arrest, we suggest that they may represent a fibroblast “quiescence program.” We further define below the characteristics of quiescent cells, in addition to cell cycle arrest, that emerge from these commonly regulated genes.

Many of the quiescence program genes have already been shown to play a role in controlling cell proliferation. The upregulated protein *MXI1* has been shown to control cell proliferation in mouse models [14]. The downregulated proteins include *FOXM1,* which has also been shown to be rate-limiting for cell proliferation in animal models [15]. Also downregulated was *CKS2* [16] and other proteins known to be associated with cell proliferation, including *cyclin B1, CENPA, thymidine kinase*, and *thymidylate synthase*. Thus, the functional relevance of the quiescence-program gene-expression changes with regard to cell-cycle arrest is clear.

Among the downregulated quiescence program genes were both *Cdc20* and *Cul-1*, indicating that quiescence involves decreased activity of two major cell-cycle, proteolytic pathways, those governed by APC *Cdc20* and the SCF. Note that a different form of the APC, APC-*Cdh1,* is activated in quiescent cells [17], suggesting that quiescence involves a switch in the regulation and function of the APC.

Many of the genes upregulated in the quiescence program are also involved in negatively controlling cell division. Indeed, some of the upregulated genes that may be involved in actively maintaining cell cycle arrest encode established and putative tumor-suppressor proteins including *MXI1, TP53, FAT,* transmembrane receptor *PTK7,* downregulated in ovarian cancer 1 *(DOC1),* tyrosine phosphatase receptor *(PTPRK),* N-myristoyltransferase 1 *(NMT1),* hypermethylated in cancer (*HIC-1*), tissue inhibitor of metalloproteinases-3 *(TIMP3),* and cadherin 11 *(CDH11).* This underscores the idea that tumorigenesis, in part, involves escape from an actively maintained nondividing state.

The genes and pathways in the fibroblast quiescence program are also important for quiescence in other cell types. Twenty-four of the genes we identified as being downregulated in the fibroblast quiescence program were also studied in T lymphocytes, and 17 of those were downregulated in quiescent versus proliferating T cells (*p* ≪ .001 by chi-squared analysis) [18] (Table 2). Moreover, 81 genes we identified as being upregulated in the fibroblast quiescence program were also studied in T lymphocytes, and 12 were expressed at higher levels in quiescent lymphocytes (*p* < .01 by chi-squared analysis). It is not unexpected that there would be less overlap among upregulated quiescence genes, because many of them participate in cell-type specific pathways such as intercellular communication and suppression of differentiation. We also highlight specific pathways that are shared between quiescent fibroblasts and T lymphocytes. One is downregulation of myc, which in fibroblasts is achieved by upregulation of the myc antagonist *MXI1,* and in T cells by transcriptional repression of the *myc* gene itself [19]. Another is modulation of the TGF-β pathway. An important component of T cell quiescence is the TGF-β agonist *TOB1* [20], and the related proteins *TOB2* and *BTG-2* are upregulated in quiescent fibroblasts.

### Quiescence Is Not Only Cell-Cycle Arrest

The genes universally expressed in quiescent fibroblasts can be broadly divided into two groups. One group includes the genes that are regulated as a downstream consequence of cell-cycle exit, for example by inhibition of CDKs. These genes are mostly repressed during cell-cycle arrest and many of them encode proteins that directly participate in cell growth and division. This suggests that feedback regulation of cell-cycle genes is an important component of exit from the cell cycle. In contrast, cell-cycle arrest was not sufficient to recapitulate the regulation of the second group of quiescence genes, which included most of the upregulated quiescence program genes. Induction of these genes was more likely to reflect the engagement of a signaling pathway shared by these extracellular arrest signals, that culminates not only in the inhibition of proliferation, but also in other gene expression changes that are required to actively maintain cells in a viable and reversible state of arrest.

### Reversible Arrest

The cardinal feature of quiescence that distinguishes it from other nondividing cell states, such as senescence, apoptosis, and terminal differentiation, is that quiescence is reversible. Some of the genes regulated in quiescent cells may, therefore, play roles in suppressing entry into these irreversible “out-of-cycle” states. In particular, we showed that both contact inhibition and serum starvation inhibited MyoD-induced myogenesis, while cells arrested by p21 overexpression entered a myogenesis pathway with an efficiency similar to controls. These results are consistent with the classical literature on myogenesis, in which it was demonstrated that cultured myoblasts can only initiate terminal myogenic differentiation when they are proliferating, whereas quiescent myoblasts are resistant to myogenic signals [21–23]. In the C2C12 myoblast cell line, MyoD-dependent differentiation involves induction of myogenin while the cells are still proliferating [24] followed by p21-dependent exit from the cell cycle [25–27]. Moreover, in vivo, quiescent myogenic stem cells (satellite cells) first proliferate in response to muscle injury before initiating myogenesis and differentiating to form new myotubes [28]. Our results extend these findings to fibroblasts and show that the block to myogenesis imposed by quiescence is at the level of responsiveness to MyoD. The p21-induced nonproliferating state remains permissive for myogenesis, which may therefore explain why the p21 mode of cell-cycle exit is utilized as part of the myogenic program.

Some of the genes regulated in quiescent cells but not cells arrested by CDK inhibition may mediate the resistance to differentiation through the Notch, bone morphogenetic protein (BMP), or Wnt pathways. The upregulated gene *HES1* (hairy and enhancer of split homolog-1), a basic helix-loop-helix (bHLH) transcriptional repressor, is a key target in Notch signaling. It blocks nerve growth factor-induced differentiation [29] and the myogenic differentiation properties of MyoD [30,31]. *SMAD1*, a signaling platform for the BMP pathway, was also consistently upregulated in quiescent cells. It can facilitate Notch signaling and thereby inhibit differentiation [32]. A number of genes in the Wnt pathway, which can suppress differentiation in stem cells [33], were upregulated in quiescent cells including *WNT2,* the frizzled receptor *FZD2, TCF7L2,* and p300/CBP-associated factor *(PCAF).* Other gene-expression changes that may play a role in suppressing differentiation include upregulation of *BCL6* [34], upregulation of matrix *Gla* protein, which inhibits chondrocyte differentiation [35], and downregulation of acyl coenzyme A binding protein *(DBI),* which promotes adipocyte differentiation [36,37]. The latter two may be particularly important in quiescent fibroblasts, which can differentiate along the chondrocyte and adipocyte lineages, whereas other types of quiescent cells may express different lineage-specific inhibitors of differentiation.

Other regulated genes may inhibit apoptosis. Upregulation of NF-kappa B family member p49/p100 *(NFKB2),* which is an inhibitor of apoptosis, was observed in cells arrested for 4 d by all signals. Cells arrested by mitogen withdrawal upregulated *MET,* which inhibits apoptosis [38], while downregulating *Bak*, an apoptosis inducer [39,40]. Cells arrested overnight by loss of adhesion upregulated *BRAG-1*, a *Bcl-2* homologue [41]. Cells arrested by contact inhibition upregulated *FYN,* which may play a role in IGF-1–mediated survival [42].

Other upregulated genes that may function to preserve the reversibility of quiescence are those that protect the cells from accumulating damage. These include superoxide dismutase-3 *(SOD3),* which scavenges free radicals [43], peroxiredoxin 4 *(PRDX4),* which protects against hydroperoxides [44], and epoxide hydrolase *(EPHX1),* which detoxifies environmental chemicals.

Surprisingly, some of the genes upregulated in the quiescence program have been previously characterized as oncogenes, including *NFKB2* [45], *BCL6,* and *cyclin D2* [47–50]. This paradox can be resolved by appreciating that tumorigenic cells share with quiescent cells the ability to suppress entry into a terminally arrested state, and therefore that oncogenic transformation should not simply be viewed as a negation of quiescence.

### Intercellular Signaling

A large number of quiescence program genes were linked to intercellular signaling (Table S1), including cell surface receptors that affect how quiescent fibroblasts respond to their environment, proteins involved in remodeling of the extracellular microenvironment, secreted proteins that could affect the proliferation of surrounding cells, and extracellular inhibitors of angiogenesis. These gene-expression changes suggest that quiescent fibroblasts may create a growth-suppressing microenvironment that affects not only other fibroblasts but also other neighboring cell types. Thus, quiescence might be an organized property of the collection of cells that comprise a tissue or organ, rather than a strictly cell-autonomous process. Fibroblasts in tumors, unlike fibroblasts in normal tissues, frequently express genes characteristic of proliferating as opposed to serum-starved, quiescent cells [51]. Tumor progression, then, could be viewed as a disruption in the quiescent state maintained in the entire tissue, and reflect changes not only in the tumor cells themselves, but also in the surrounding stroma and the vasculature.

## Materials and Methods

### Cells

Fetal human lung diploid fibroblasts from American Type Culture Collection (CCL153) were grown in FBM medium (Biowhittaker, Walkersville, Maryland, United States) supplemented with 2% fetal calf serum, 5 μg/ml insulin, and 1 ng/ml fibroblast growth factor (FGM medium). Cells were grown at 37 °C in 5% CO_2_ and routinely split 1 to 3 every 3 d. For each condition, frozen aliquots of 5 × 10^6^ cells were expanded for five passages, and approximately 4 × 10^7^ cells were plated in the given condition.

### Arrest conditions

For growing cultures, cells were plated at a density of 2 × 10^4^ cells per cm^2^ in FGM medium and allowed to attach to the tissue culture plate. Because we wanted to explore the effects of mitogen withdrawal on the cell cycle without the many other effects of changes in serum concentrations, cells were grown or arrested in low-serum conditions in the presence or absence of the mitogen platelet-derived growth factor (PDGF). The medium was changed to FBM with 0.1% fetal calf serum, 25 ng/ml IGF-1 (R&D Systems, Minneapolis, Minnesota, United States), and 30 ng/ml PDGF (R&D Systems). Cells arrested by mitogen withdrawal were plated at a concentration of 10^4^ cells/cm^2^ and allowed to attach to the tissue culture plate. The medium was changed to FBM, 0.1% serum, and IGF-1. To arrest cells by loss of adhesion, tissue culture plates were coated with FBM medium containing 1% agarose that was allowed to solidify to prevent cells from attaching to the plate. Cells were placed in a suspension of FBM, 0.1% serum, IGF-1 and PDGF supplemented with 0.4% methylcellulose (Fisher Scientific, Fair Lawn, New Jersey, United States). For cells arrested by contact inhibition, the cells were plated at 2 × 10^5^ cells/cm^2^ and allowed to attach before the medium was changed to 0.1% serum, IGF-1 and PDGF. Cells were incubated either for 14 h or for 4 d with medium changes every 2 d. For arrest by growth to confluence, cells were plated at a density of 2 × 10^4^ cells per cm^2^ in FGM and allowed to grow to confluence. Medium was changed every 2 to 3 d for 4 d or approximately 20 (17–22) d. The night before cells were collected for flow cytometry, the medium was changed to FBM, 0.1% serum, IGF-1 and PDGF. For each condition, two independent replicates were performed.

### Arrest via CKI overexpression

To arrest CCL153 by CKI overexpression, Phoenix cells (American Type Culture Collection, Manassas, Virginia, United States) were transfected with QCXIP empty vector or QCXIP into which the coding sequence of p21 or p27 had been introduced with fugene (Roche, Indianapolis, Indiana, United States). Due to a change in the termination codon, the p21 vector expresses a protein that is 6-kDa larger than p21 at its C terminus. The viral supernatants from transduced Phoenix cells were collected 48 h later and used to transduce fibroblasts. Two days after transduction, the cells were selected with 5 μg/ml puromycin for 2 d. The night before cells were collected for flow cytometry, the medium was changed to FBM, 0.1% serum, IGF-1 and PDGF.

### Bromodeoxyuridine labeling

Cells were labeled with 100-μM bromodeoxyuridine (BrdU) for 6 h. Cells were collected with trypsin. The trypsin was inactivated with serum, and cells were fixed in PBS with 67% cold ethanol. Cell membranes were lysed at 37 °C in 0.08% pepsin for 20 min, and nuclei were treated with 2M HCl for 20 min. Samples were neutralized with 0.1M sodium borate, then incubated in a buffer of 10 mM Hepes, (pH7.4), 150 mM NaCl, 4% fetal bovine serum, and 2 μg of anti-BrdU-FITC antibody (Pharmingen, San Diego, California, United States) on ice for 2 h. The antibody was removed and cells were incubated in the same buffer with 0.5% Tween-20, 250 μg/ml RNase A, 100 μg/ml propidium iodide, and analyzed on a Beckton Dickinson FACS Calibur (Becton Dickinson, Palo Alto, California, United States).

### Flow cytometry

Cells were incubated with approximately 5 μg/ml Hoescht 33342 (Calbiochem-Novabiochem, San Diego, California, United States) for 45 min. Cells were treated with trypsin and collected into 1 mg/ml soybean trypsin inhibitor (Sigma, St. Louis, Missouri, United States), centrifuged and resuspended in Hanks balanced saline solution supplemented with BSA (100 mg/ml), gelatin (0.04%), and EGTA (5M). Propidium iodide (0.5 μg/ml) was added, and the cells were incubated on ice. For cells arrested by mitogen withdrawal for 4 d, Hoescht was not applied because it induced cell death. Samples were run on a Beckton Dickinson FACS Vantage SE.

### Microarray analysis

RNA was converted into target and hybridized to HuGENE-FL microarrays as described previously [52]. Microarray results were analyzed with the GENECHIP software (Affymetrix suite 4.0 or 5.0, Affymetrix, Santa Clara, California, United States). Each chip was scaled such that the average intensity was 1,000 while masking outliers (suite 4.0) or 500 (suite 5.0). Because of differences in the overall intensity of the different chips used in these experiments, only genes with average difference values in the first growing sample between ten and 6,700 were considered for analysis. This resulted in the systematic exclusion of classes of proteins expressed at high levels, for instance, ribosomal proteins.

### Neighbor-joining trees

The transcriptional distance between two samples was determined for each pair of samples as the sum of the log base two of the absolute values of the fold changes for each gene, or the sum of the signal log ratios. This value was assigned a zero for a particular gene if there was a “no change” call between the samples or if the gene was absent in both samples. The distance matrix was used to create neighbor-joining trees with the Phylip software suite version 3.5 (available at http://evolution.genetics.washington.edu/phylip/software.html).

### Identifying regulated genes

In the first method, we required that all of the gene expression values were higher or lower in the regulated samples as compared with all samples in which the gene was not regulated. We then required that the change in gene expression met a 2-fold cutoff under regulated conditions but did not change 2-fold in unregulated conditions. 2-fold cutoffs were assessed by comparing arrested state replicate 1 to growing sample replicate 1 and arrested state replicate 2 to growing sample replicate 2. To avoid dividing by small or negative numbers, average difference values less than 20 were thresholded to a value of 20. The number of genes expected by chance was determined by performing the same analysis on the same data set with permuted column headings. For prolonged arrest, we only used the 2-fold cutoff approach.

In the second method, we used a template-matching approach described in more detail previously [52]. An idealized template vector consisting of zeroes and ones was compared with the vector of average difference values for all of the genes that passed the filter, and the Pearson correlation coefficient was computed [53]. Genes that matched the template better than was ever observed in 20 permutations of the entire data set (0.05 genes expected) were included in the list of consistently regulated genes.

The total number of false positives expected (the sum of the number expected from both methods) was compared with the observed number of regulated genes based on a Poisson distribution.

Defining a quiescence program: the expression values in all 4-d or longer samples were required to be higher or lower than the values in the growing samples or the growing samples with an empty vector. In permuted data, 1.4 upregulated and 3.4 downregulated genes met these criteria. In contrast, 205 genes were expressed at a higher level in all longer arrested states, and 59 genes were expressed at a lower level in all longer arrested states. We then required that the gene expression value in each of the longer arrested states was consistently 2-fold higher than in growing cells. There were 43 upregulated genes that met these criteria and 16 downregulated genes, whereas 0.1 upregulated and 0.1 downregulated genes were expected by chance.

To this list of consistently regulated genes, we added genes that met the template-matching criteria for eight different templates. The templates were defined by higher (or lower) expression in all arrests for 4 or 20 d, and all combinations of regulation in the same direction in subsets of overnight samples. For each template, genes with Pearson correlation coefficients closer to −1 or 1 than observed for any gene when the entire data set was permuted 20× were included. These eight different template-matching tests resulted in a total of 98 upregulated genes and 30 downregulated genes when 0.4 (8 × .05) genes were expected by chance. The total number of quiescence program genes when the two lists were merged was 116 upregulated and 33 downregulated.

### Myogenesis

For myogenesis experiments, Phoenix cells were transfected with pBabe-MyoD-ER, and the supernatant was used to transduce human dermal fibroblasts 91-SF5 (generous gift of T. Norwood, University of Washington, Seattle, Washington, United States), as described above. 91-SF5 cells were grown in phenol red-free DMEM, supplemented with 10% bovine growth serum (Hyclone, Logan, Utah, United States), and were selected with 2 μg/ml puromycin for 2 d. To induce differentiation, 91-SF5 cells transduced with pBabe-MyoD-ER were washed twice with PBS and the medium was changed to phenol-red free DMEM with insulin (10 μg/ml, Sigma), transferrin (10 μg/ml, Sigma), horse serum (0.5%, or 0.2% for serum-starved samples, Gibco/Invitrogen, Carlsbad, California, United States), and β-estradiol (10^−7^ M; Sigma). For serum-starved samples, cells were incubated in phenol red-free DMEM with no serum for 4 d prior to differentiation. For contact-inhibited samples, cells were plated at high density (2 × 10^5^ cells/cm^2^) and incubated for 4 d prior to differentiation. For CDK inhibition, 91-SF5 cells were transduced with a pBabe-MyoD-ER vector containing a gene-encoding hygromycin resistance, and selected with hygromycin (250 μg/ml). Cells were then transduced with either the QCXIP empty vector or QCXIP containing the p21 coding sequence, and selected with puromycin (2 μg/ml). Cells were then induced to differentiate. Samples were collected into Trizol reagent at the indicated times for real-time RT-PCR.

For real-time RT-PCR, RNA was isolated as described above. cDNA was synthesized from 2 μg of total RNA using Superscript II reverse transcriptase (Invitrogen) primed with poly-T oligonucleotides according to the manufacturer's instructions. One-tenth the final volume was used for real-time PCR with master-mix–containing primers and probes specific for myosin heavy chain (ID number Hs00428600_m1, Applied Biosystems, Foster City, California, United States). Real-time PCR was performed on an ABI Prism 7900 instrument. Samples were normalized for expression levels of human GAPDH (Applied Biosystems).

## Supporting Information

Figure S1 Persistence of Gene Expression Changes from 14 h to 4 d Using template-matching, the number of genes upregulated and downregulated by mitogen withdrawal (A), contact inhibition (B), and loss of adhesion (C) was determined based on a Pearson correlation coefficient threshold of two genes by chance. For each arrest signal, the number of genes regulated by only an overnight arrest, by both overnight and 4 d, and only 4 d was determined. A signature was considered present if more than seven genes were included. Data are normalized and depicted as heat maps generated with Java Treeview.
(2.2 MB PDF).

Figure S2 Genes Regulated in Opposite Directions by Mitogen Withdrawal and Loss of Adhesion Genes upregulated by mitogen withdrawal and downregulated by loss of adhesion, and vice versa, were identified by template-matching using a threshold of two genes by chance. The signatures at quiescence initiation and quiescence maintenance are shown as heat maps.
(2.6 MB TIF).

Table S1 Quiescence Genes Involved in Intercellular Communication Genes regulated by one, two, or three signals at 14 h, 4 d, or 20 d that are involved in intercellular communication are listed, along with their classification by functional category.
(232 KB DOC).

## Abbreviations

BMP: bone morphogenetic protein
BrdU: bromodeoxyuridine
CDK: cyclin-dependent kinase
CKI: cyclin-dependent kinase inhibitor
ER: estrogen receptor
GO: gene ontology
IGF: insulin-like growth factor
PDGF: platelet-derived growth factor
