# Supplementary material for: A New Description of Cellular Quiescence
Source: PLoS Biol. 2006 Mar 7;4(3):e83. doi: 10.1371/journal.pbio.0040083 (PMC1393757; doi:10.1371/journal.pbio.0040083)
Supplement: Supplementary file 1 [file pbio.0040083.sg001.pdf]

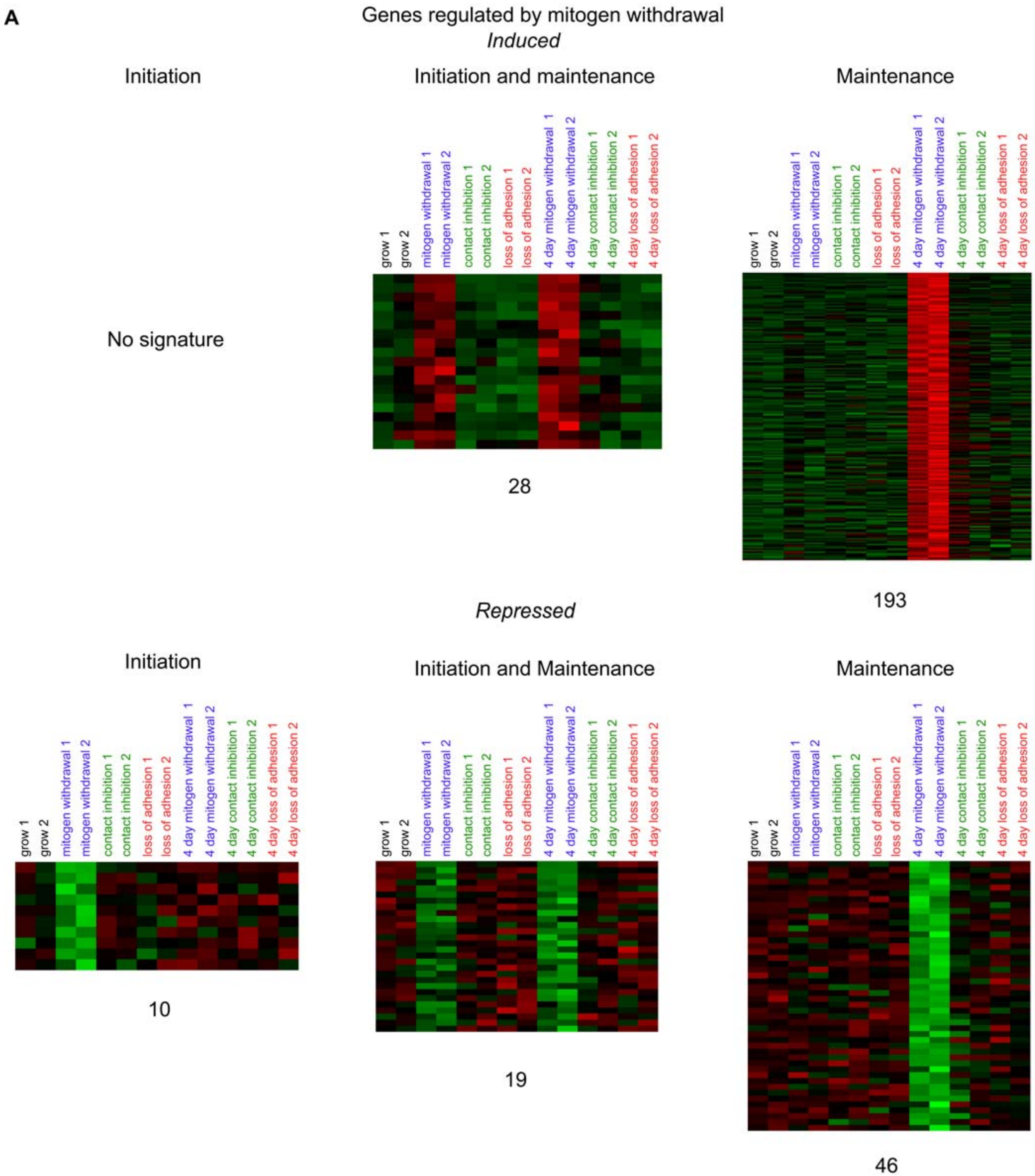
A
Genes regulated by mitogen withdrawal
Induced
Initiation
No signature
Initiation and maintenance
28
Maintenance
193
Repressed
Initiation
10
Initiation and Maintenance
19
Maintenance
46
grow 1
grow 2
mitogen withdrawal 1
mitogen withdrawal 2
contact inhibition 1
contact inhibition 2
loss of adhesion 1
loss of adhesion 2
4 day mitogen withdrawal 1
4 day mitogen withdrawal 2
4 day contact inhibition 1
4 day contact inhibition 2
4 day loss of adhesion 1
4 day loss of adhesion 2

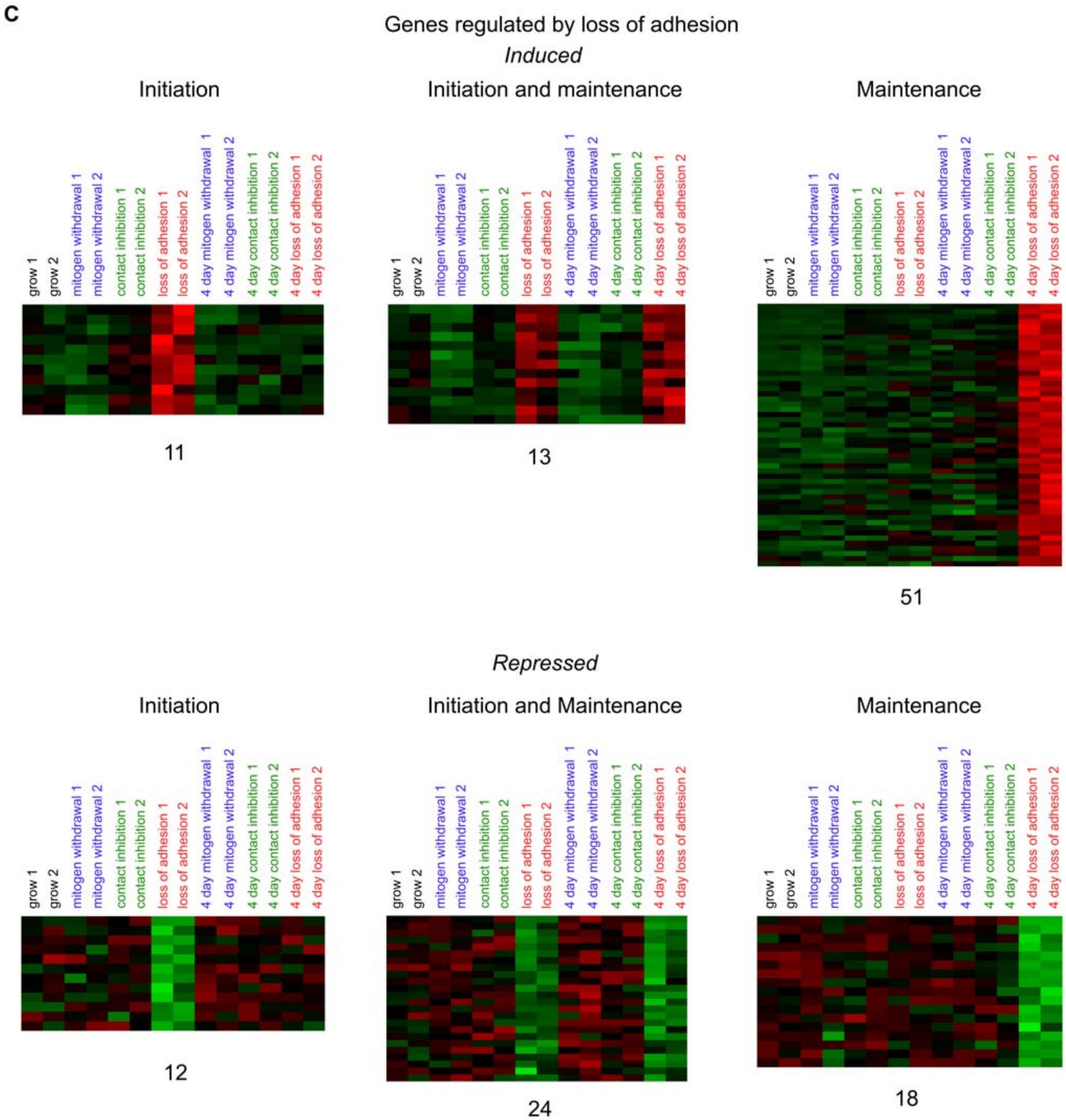
C
Genes regulated by loss of adhesion
Induced
Initiation
Initiation and maintenance
Maintenance
grow 1
grow 2
mitogen withdrawal 1
mitogen withdrawal 2
contact inhibition 1
contact inhibition 2
loss of adhesion 1
loss of adhesion 2
4 day mitogen withdrawal 1
4 day mitogen withdrawal 2
4 day contact inhibition 1
4 day contact inhibition 2
4 day loss of adhesion 1
4 day loss of adhesion 2
11
13
51
Repressed
Initiation
Initiation and Maintenance
Maintenance
12
24
18

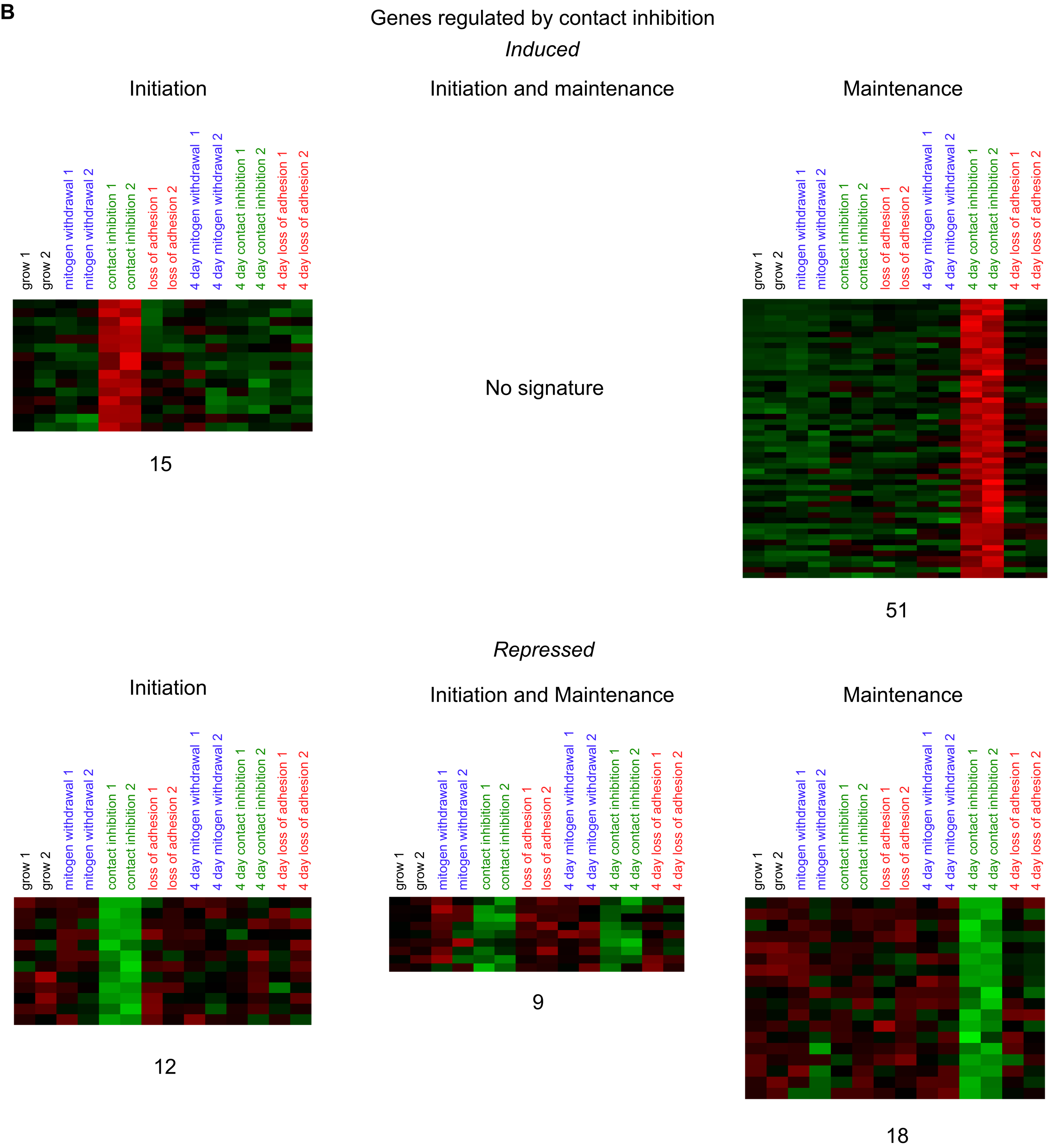
B
Genes regulated by contact inhibition
Induced
Initiation
Initiation and maintenance
Maintenance
grow 1
grow 2
mitogen withdrawal 1
mitogen withdrawal 2
contact inhibition 1
contact inhibition 2
loss of adhesion 1
loss of adhesion 2
4 day mitogen withdrawal 1
4 day mitogen withdrawal 2
4 day contact inhibition 1
4 day contact inhibition 2
4 day loss of adhesion 1
4 day loss of adhesion 2
15
No signature
51
Repressed
Initiation
Initiation and Maintenance
Maintenance
12
9
18
