# Supplementary material for: A New Description of Cellular Quiescence
Source: PLoS Biol. 2006 Mar 7;4(3):e83. doi: 10.1371/journal.pbio.0040083 (PMC1393757; doi:10.1371/journal.pbio.0040083)
Supplement: Supplementary file 2 [file pbio.0040083.st001.doc]

| Table 1: Genes regulated by specific arrest signals | | | | | |
| --- | --- | --- | --- | --- | --- |
| Gene name | GO terms | Mitogen Withdrawal | Contact Inhibition | Loss of Adhesion | 20 dy Confluence |
| 14 hours |  |  |  |  |  |
| DCN: decorin | extracellular matrix | ⏶ |  |  |  |
| BDNF: brain-derived neurotrophic factor | secreted signaling | ⏶ |  |  |  |
| CXCL2: chemokine (C-X-C motif) ligand 2 | secreted signaling | ⏶ |  |  |  |
| GDF5: growth differentiation factor 5 | secreted signaling | ⏶ |  |  |  |
| IGFBP3: insulin-like growth factor binding protein 3 | secreted signaling | ⏶ |  |  |  |
| WNT2: wingless-type MMTV integration site family member 2 | secreted signaling | ⏶ |  |  |  |
| SERPINF1: serpin peptidase inhibitor, clade F | angiogenesis inhibition |  | ⏶ |  |  |
| ITGA8: integrin, alpha 8 | cell surface protein |  | ⏶ |  |  |
| CALCRL:  calcitonin receptor-like | cell surface receptor |  | ⏶ |  |  |
| GHRHR: growth hormone releasing hormone receptor | cell surface receptor |  | ⏶ |  |  |
| IL6: interleukin 6 | secreted signaling |  | ⏶ |  |  |
| IL2RG: interleukin 2 receptor, gamma | cell surface receptor |  |  | ⏶ |  |
| NPR3: natriuretic peptide receptor C/guanylate cyclase C | cell surface receptor | ⏶ | ⏶ |  |  |
| AR: androgen receptor | cell surface receptor |  | ⏶ | ⏶ |  |
| GRIA4: glutamate receptor, ionotrophic, AMPA 4 | cell surface receptor | ⏶ | ⏶ | ⏶ |  |
| ITGA2: integrin, alpha 2 | cell surface protein | ⏷ |  |  |  |
| PLAUR: plasminogen activator, urokinase receptor | cell surface receptor | ⏷ |  |  |  |
| COL13A1: collagen, type XIII, alpha 1 | extracellular matrix | ⏷ |  |  |  |
| MFAP2: microfibrillar-associated protein 2 | extracellular matrix | ⏷ |  |  |  |
| MMP10: matrix metallopeptidase 10 | extracellular matrix | ⏷ |  |  |  |
| NOS2A: nitric oxide synthase 2A | nitric oxide signaling | ⏷ |  |  |  |
| STC1:  stanniocalcin 1 | secreted protein | ⏷ |  |  |  |
| IL11: interleukin 11 | secreted signaling | ⏷ |  |  |  |
| NPPA: natriuretic peptide precursor A | secreted signaling | ⏷ |  |  |  |
| PLAT: plasminogen activator, tissue | secreted signaling | ⏷ |  |  |  |
| PLAU: plasminogen activator, urokinase | secreted signaling | ⏷ |  |  |  |
| SEMA3A: semophorin 3A | secreted signaling | ⏷ |  |  |  |
| FGF4: fibroblast growth factor 4 | secreted signaling |  |  | ⏷ |  |
| SEMA3B: semaphorin 3B | secreted signaling |  |  | ⏷ |  |
| HAS2: hyaluronan synthase 2 | extracellular matrix | ⏷ | ⏷ |  |  |
| FGF5: fibroblast growth factor 5 | secreted signaling |  | ⏷ | ⏷ |  |
| VEGFC: vascular endothelial growth factor C | angiogenesis | ⏷ | ⏷ | ⏷ |  |
|  |  |  |  |  |  |
| Four days |  |  |  |  |  |
| ANGPT1: angiopoietin 1 | angiogenesis | ⏶ |  |  |  |
| CYR61: cysteine-rich, angiogenic inducer, 61 | angiogenesis | ⏶ |  |  |  |
| ERBB3: v-erb-b2 erythroblastic leukemia viral oncogene homolog 3 | cell surface receptor | ⏶ |  |  |  |
| ESR2: estrogen receptor 2 | cell surface receptor | ⏶ |  |  |  |
| IL6R: interleukin 6 receptor | cell surface receptor | ⏶ |  |  |  |
| THRB: thyroid hormone receptor, beta | cell surface receptor | ⏶ |  |  |  |
| BMP4: bone morphogenetic protein 4 | secreted signaling | ⏶ |  |  |  |
| CXCL2: chemokine (C-X-C motif) ligand 2 | secreted signaling | ⏶ |  |  |  |
| FGF2: fibroblast growth factor 2 (basic) | secreted signaling | ⏶ |  |  |  |
| PTHLH: parathyroid hormone-like hormone | secreted signaling | ⏶ |  |  |  |
| SEMA3F: semaphorin 3F | secreted signaling | ⏶ |  |  |  |
| TGFB2: transforming growth factor, beta 2 | secreted signaling | ⏶ |  |  |  |
| TGFB3: transforming growth factor, beta 3 | secreted signaling | ⏶ |  |  |  |
| WNT2: wingless-type MMTV integration site family member 2 | secreted signaling | ⏶ |  |  |  |
| ITGB5: integrin, beta 5 | cell surface adhesion molecule |  | ⏶ |  |  |
| CXADR: coxsackie virus and adenovirus receptor | cell surface receptor |  |  | ⏶ |  |
| COL4A2: collagen, type IV, alpha 2 | extracellular matrix |  |  | ⏶ |  |
| COL8A1: collagen, type VIII, alpha 1 | extracellular matrix |  |  | ⏶ |  |
| ECM1: extracellular matrix protein 1 | extracellular matrix |  |  | ⏶ |  |
| MMP11: matrix metallopeptidase 11 | extracellular matrix |  |  | ⏶ |  |
| MMP3: matrix metallopeptidase 3 | extracellular matrix |  |  | ⏶ |  |
| HTRA1: HtrA serine peptidase 1 | secreted signaling |  |  | ⏶ |  |
| THBS2: thrombospondin 2 | angiogenesis inhibition | ⏶ | ⏶ |  |  |
| CDH13: cadherin 13, H-cadherin | cell surface protein | ⏶ | ⏶ |  |  |
| FZD2: frizzled homolog 2 | cell surface receptor | ⏶ | ⏶ |  |  |
| HTR2B: 5-hydroxytryptamine (serotonin) receptor 2B | cell surface receptor | ⏶ | ⏶ |  |  |
| IGF2R: insulin-like growth factor 2 receptor | cell surface receptor | ⏶ | ⏶ |  |  |
| TGFBR3: transforming growth factor, beta receptor III | cell surface receptor | ⏶ | ⏶ |  |  |
| COL15A1: collagen, type XV, alpha 1 | extracellular matrix | ⏶ | ⏶ |  |  |
| DCN: decorin | extracellular matrix | ⏶ | ⏶ |  |  |
| FBN1: fibrillin 1 | extracellular matrix | ⏶ | ⏶ |  |  |
| LAMA4: laminin, alpha 4 | extracellular matrix | ⏶ | ⏶ |  |  |
| MFAP1: microfibrillar-associated protein 1 | extracellular matrix | ⏶ | ⏶ |  |  |
| BDNF: brain-derived neurotrophic factor | secreted signaling | ⏶ | ⏶ |  |  |
| WNT2: wingless-type MMTV integration site family member 2 | secreted signaling | ⏶ | ⏶ |  |  |
| COL1A1: collagen, type I, alpha 1 | extracellular matrix | ⏶ |  | ⏶ |  |
| LAMB2: laminin, beta 2 | extracellular matrix | ⏶ |  | ⏶ |  |
| TNC: tenascin C | extracellular matrix | ⏶ |  | ⏶ |  |
| NPPB: natriuretic peptide precursor B | angiogenesis inhibition | ⏶ |  | ⏶ |  |
| ITGB5: integrin, beta 5 | cell surface protein |  | ⏶ | ⏶ |  |
| COL13A1: collagen, type XIII, alpha 1 | extracellular matrix |  | ⏶ | ⏶ |  |
| MMP11: matrix metallopeptidase 11 | extracellular matrix |  | ⏶ | ⏶ |  |
| SERPINF1: pigment epithelium derived factor | angiogenesis inhibition | ⏶ | ⏶ | ⏶ |  |
| TIMP3: tissue inhibitor of metalloproteinase 3 | angiogenesis inhibition | ⏶ | ⏶ | ⏶ |  |
| AGT: angiotensinogen (serine (or cysteine) proteinase inhibitor) | angiogenesis inhibition | ⏶ | ⏶ | ⏶ |  |
| CDH11: cadherin 11, type 2 | cell surface protein | ⏶ | ⏶ | ⏶ |  |
| FAT: FAT tumor suppressor homolog 1 | cell surface protein | ⏶ | ⏶ | ⏶ |  |
| ITGA1: integrin, alpha 1 | cell surface protein | ⏶ | ⏶ | ⏶ |  |
| VCAM1: vascular cell adhesion molecule 1 | cell surface protein | ⏶ | ⏶ | ⏶ |  |
| BDKRB2: bradykinin receptor B2 | cell surface receptor | ⏶ | ⏶ | ⏶ |  |
| ERBB2: v-erb-b2 erythroblastic leukemia viral oncogene homolog 2 | cell surface receptor | ⏶ | ⏶ | ⏶ |  |
| FCGRT: Fc fragment of IgG, receptor, transporter, alpha | cell surface receptor | ⏶ | ⏶ | ⏶ |  |
| FGFR1: fibroblast growth factor receptor 1 | cell surface receptor | ⏶ | ⏶ | ⏶ |  |
| GPR37: G protein-coupled receptor 37 | cell surface receptor | ⏶ | ⏶ | ⏶ |  |
| IL1R1: interleukin 1 receptor, type I | cell surface receptor | ⏶ | ⏶ | ⏶ |  |
| LRP1: low density lipoprotein-related protein 1 | cell surface receptor | ⏶ | ⏶ | ⏶ |  |
| OBRGRP: leptin receptor gene-related protein | cell surface receptor | ⏶ | ⏶ | ⏶ |  |
| PTPRG: protein tyrosine phosphatase, receptor type, G | cell surface receptor | ⏶ | ⏶ | ⏶ |  |
| ROR1: receptor tyrosine kinase-like orphan receptor 1 | cell surface receptor | ⏶ | ⏶ | ⏶ |  |
| TFRC: transferrin receptor | cell surface receptor | ⏶ | ⏶ | ⏶ |  |
| TGFBR2: transforming growth factor, beta receptor II | cell surface receptor | ⏶ | ⏶ | ⏶ |  |
| TNFRSF1A: tumor necrosis factor receptor superfamily, member 1A | cell surface receptor | ⏶ | ⏶ | ⏶ |  |
| A2M: alpha-2-macroglobulin | extracellular matrix | ⏶ | ⏶ | ⏶ |  |
| COL3A1: collagen, type III, alpha 1 | extracellular matrix | ⏶ | ⏶ | ⏶ |  |
| COL4A5: collagen, type IV, alpha 5 | extracellular matrix | ⏶ | ⏶ | ⏶ |  |
| LAMC1: laminin, gamma 1 | extracellular matrix | ⏶ | ⏶ | ⏶ |  |
| LUM: lumican | extracellular matrix | ⏶ | ⏶ | ⏶ |  |
| MGP: matrix Gla protein | extracellular matrix | ⏶ | ⏶ | ⏶ |  |
| TNC: tenascin C (hexabrachion) | extracellular matrix | ⏶ | ⏶ | ⏶ |  |
| BMP1: bone morphogenetic protein 1, (pro collagen C proteinase) | secreted protein | ⏶ | ⏶ | ⏶ |  |
| C1R: complement component 1, r subcomponent | secreted protein | ⏶ | ⏶ | ⏶ |  |
| C1S: complement component 1, s subcomponent | secreted protein | ⏶ | ⏶ | ⏶ |  |
| CFHL1: complement factor H-related 1 | secreted protein | ⏶ | ⏶ | ⏶ |  |
| SOD3: superoxide dismutase 3, extracellular | secreted protein | ⏶ | ⏶ | ⏶ |  |
| F3: coagulation factor III (thromboplastin, tissue factor) | secreted signaling | ⏶ | ⏶ | ⏶ |  |
| IL6: interleukin 6 | secreted signaling | ⏶ | ⏶ | ⏶ |  |
| PLAT: plasminogen activator, tissue | secreted signaling | ⏶ | ⏶ | ⏶ |  |
| PROS1: protein S (alpha) | secreted signaling | ⏶ | ⏶ | ⏶ |  |
| SERPING1: serine (or cysteine) proteinase inhibitor | secreted signaling | ⏶ | ⏶ | ⏶ |  |
|  |  |  |  |  |  |
| ITGA2: integrin, alpha 2 | cell surface adhesion molecule | ⏷ |  |  |  |
| CXADR: coxsackie virus and adenovirus receptor | cell surface receptor | ⏷ |  |  |  |
| PLAUR: plasminogen activator, urokinase receptor | cell surface receptor | ⏷ |  |  |  |
| TGFB1: transforming growth factor, beta 1 | secreted signaling | ⏷ |  |  |  |
| BMP3: bone morphogenetic protein 3 | secreted signaling |  | ⏷ |  |  |
| EDG2: endothelial differentiation, lysophosphatidic acid receptor, 2 | cell surface receptor |  |  | ⏷ |  |
| LDLR: low density lipoprotein receptor | cell surface receptor |  |  | ⏷ |  |
| FGF5: fibroblast growth factor 5 | secreted signaling |  |  | ⏷ |  |
| TFPI2: tissue factor pathway inhibitor 2 | extracellular matrix | ⏷ | ⏷ |  |  |
| NOS2A: nitric oxide synthase 2A | nitric oxide synthesis | ⏷ | ⏷ |  |  |
| DLG7: discs, large homolog 7 (Drosophila) | cell surface protein | ⏷ | ⏷ | ⏷ |  |
| OPRK1: opioid receptor, kappa 1 | cell surface receptor | ⏷ | ⏷ | ⏷ |  |
|  |  |  |  |  |  |
| 20 days |  |  |  |  |  |
| FAS: Fas (TNF receptor superfamily, member 6) | cell surface receptor |  |  |  | ⏶ |
| IL32: interleukin 32 | secreted signaling |  |  |  | ⏶ |
|  |  |  |  |  |  |
| CSPG4: chondroitin sulfate proteoglycan 4 | cell surface protein |  |  |  | ⏷ |
| ITGA9: integrin, alpha 9 | cell surface protein |  |  |  | ⏷ |
| BMPR1A: bone morphogenetic protein receptor | cell surface receptor |  |  |  | ⏷ |
| LIFR: leukemia inhibitory factor receptor | cell surface receptor |  |  |  | ⏷ |
| PGRMC1: progesterone receptor membrane component 1 | cell surface receptor |  |  |  | ⏷ |
| COL6A2: collagen, type VI, alpha 2 | extracellular matrix |  |  |  | ⏷ |
| MMP14: matrix metallopeptidase 14 | extracellular matrix |  |  |  | ⏷ |
| C8A: complement component 8, alpha polypeptide | secreted protein |  |  |  | ⏷ |
| GDF1: growth differentiation factor 1 | secreted signaling |  |  |  | ⏷ |
| IL13: interleukin 13 | secreted signaling |  |  |  | ⏷ |
| PLAU: plasminogen activator, urokinase | secreted signaling |  |  |  | ⏷ |
